# Development and Validation of the Particle into Nitroxide Quencher System with BPEAnit Probe for High-Sensitivity Reactive Oxygen Species Detection in Atmospheric Monitoring

**DOI:** 10.3390/s25041129

**Published:** 2025-02-13

**Authors:** Ruiwen Wang, Jiawen Li, Hao Wang, Shuo Deng, Congrong He, Branka Miljevic, Zoran Ristovski, Boguang Wang

**Affiliations:** 1 College of Environment and Climate, Jinan University, Guangzhou 511443, China; 2 School of Earth and Atmospheric Sciences, International Laboratory for Air Quality and Health, Queensland University of Technology, Brisbane, QLD 4000, Australia; 3 JNU-QUT Joint Laboratory for Air Quality Science and Management, Jinan University, Guangzhou 511443, China; 4 Innovation Base for Air Quality Science and Management for Guangdong, Hong Kong and Macao Greater Bay Area, Jinan University, Guangzhou 511443, China

**Keywords:** reactive oxygen species, BPEAnit, PINQ, standardization, detection sensitivity, atmospheric monitoring

## Abstract

Reactive oxygen species (ROS) play an important role in atmospheric pollution, and their detection is essential for assessing air quality and health risks. This study developed and validated a standardized methodology for using the BPEAnit probe in a specially designed particle-into-liquid sampler, the Particle Into Nitroxide Quencher (PINQ), to measure reactive oxygen species in atmospheric monitoring applications. The method demonstrated high sensitivity, with a detection limit of 0.03 nmol·m^−3^, robust linearity (R^2^ = 0.9999), and negligible system residue, ensuring accurate ROS quantification. Comparative analyses of startup conditions revealed superior baseline stability under cold start conditions despite the longer stabilization time required. The auto-oxidation of the BPEAnit probe, measured at a rate of 3.01 nmol·m^−3^ per hour, was identified as a critical factor for long-term monitoring, highlighting the necessity of standardized procedures to mitigate the drift effect. The study established the system’s suitability for urban air quality assessments and public health risk evaluations, offering insights into its limitations and operational challenges. Future advancements could focus on enhancing probe stability and expanding the method’s utility in diverse operational environments, thereby broadening its applicability to diverse monitoring scenarios.

## 1. Introduction

Reactive oxygen species (ROS) are critical atmospheric pollutants, including ·OH, ^1^O_2_, O_2_^−^·, RO·, ROO·, H_2_O_2_, ROOH, etc., which significantly influence air quality, atmospheric chemistry, and public health [1,2,3]. As reactive intermediates, ROS play a key role in atmospheric oxidation processes, contributing to the formation of secondary pollutants such as ozone and particulate matter (PM) [4,5]. The majority of ROS are generated through the photolysis of ozone, followed by subsequent free radical reactions [6,7]. Anthropogenic activities, such as vehicle emissions, industrial discharges, and biomass combustion, also contribute to ROS levels in the atmosphere [8,9,10,11,12]. Studies have shown that a significant amount of ROS is present in both indoor and outdoor air [13,14]. The health implications of ROS exposure are profound, as they are directly linked to the pathogenesis of respiratory and cardiovascular diseases [15,16]. ROS could induce oxidative stress, causing damage to cellular structures and biological macromolecules in human tissues [17]. Therefore, the precise quantification of atmospheric ROS is essential for pollution source characterization, understanding oxidative mechanisms, and developing effective public health strategies to mitigate pollution-related health impacts.

The most commonly applied methodologies for the detection of ROS fall into two main categories. The first is direct measurement, which detects ROS physically present on the surface or within PM. A widely used probe for this purpose is 2′,7′-dichlorofluorescin diacetate (DCFH), as highlighted in several studies [18,19]. The second category involves indirect measurement, which evaluates the oxidative potential (OP) of PM. Oxidative potential reflects the capacity of PM to induce oxidative stress in biological systems and is proportional to the generation rate of ROS [20]. Among acellular assays, the dithiothreitol (DTT) method is the most widely used [21,22]. Despite their widespread use in environmental research, these methods have notable limitations. Both DCFH and DTT assays suffer from probe instability and susceptibility to interference, leading to significant variability in reproducibility across studies [11,23,24]. Particularly, the common issue of these methods is a prolonged sample reaction time, which is highly unfavorable for the real-time measurement of short-lived ROS [25,26,27]. Another noteworthy point is that there is a lack of standardized procedures across different methods, and even within the same method, operational conditions and workflows vary considerably [1,28]. These apparent methodological limitations create a need for the elaboration of more reliable and sensitive detection techniques that would realistically reflect ROS dynamics in varied atmospheric conditions.

In response to the limitations of the traditional ROS detection methods, a promising alternative involves the use of 9,10-bis-(phenylethynyl)anthracene-nitroxide (BPEAnit), which offers considerable advantages over conventional assays [29]. BPEAnit in DMSO is sensitive to ROS, including ·OH, ROO·, C-centered radicals, etc. For example, DMSO in the probe rapidly reacts with ·OH to generate methyl radicals. These highly reactive methyl radicals interact with the BPEAnit probe, forming a stable fluorescent derivative, BPEAnit-Me [30,31]. The whole reaction is diffusion-limited, which enables rapid quantification and heightened sensitivity [30,32]. Currently, real-time measurement has become the key direction for ROS research and development [13,33]. Unlike DTT and DCFH-DA, BPEAnit’s shorter reaction time makes it highly suitable for the trace-level detection of ROS in real-time ambient air monitoring. When combined with the Particle Into Nitroxide Quencher (PINQ), BPEAnit facilitates continuous, real-time ROS measurements with a time resolution of just one minute [25]. The PINQ system demonstrates excellent collection efficiency for PM with diameters of 20 nmol or larger, enhancing the reliability of ROS detection. BPEAnit and PINQ hold significant potential for advancing our understanding of atmospheric oxidation processes and their implications for air quality and public health. Despite its advantages, the combined BPEAnit-PINQ system requires extensive performance evaluation across diverse environmental scenarios. Factors such as system stability, probe sensitivity, and variability may impact the accuracy and reliability of ROS detection. Systematic studies are essential to assess the robustness of BPEAnit under standardized application and to address the potential limitations that could affect its broader application in routine environmental monitoring.

This study aims to systematically evaluate the utility of BPEAnit within the PINQ system for standardized ROS measurement. Laboratory experiments focus on assessing sensitivity, reproducibility, and operational feasibility under different conditions. The research also seeks to address gaps in the existing methodologies by providing a more reliable approach for ROS monitoring. By standardizing the use of the PINQ system, this study enhances the credibility of the results and facilitates cross-comparisons among different experiments. In the long term, the findings are expected to inform better practices for ROS monitoring, broadening its application and advancing the understanding of ROS dynamics in the atmosphere.

## 2. Materials and Methods

### 2.1. Probes and Materials

A 250 nmol stock solution of the BPEAnit probe, synthesized by JICE Instrument Co. (Zhuhai, China), was prepared using dimethyl sulfoxide (DMSO, AR grade, Macklin, Shanghai, China). For system calibration, BPEAnit-Me (the methylated product of BPEAnit, synthesized by JICE Instrument Co.) was utilized. The stock solution was sequentially diluted with DMSO to prepare standard solutions at concentrations of 5, 10, 20, 50, 75, 100, 150, and 200 nmol. All the solutions were mixed thoroughly using rotators (Scilogex MX-S and MX-RD-E, Rocky Hill, CT, USA) for 30 min to ensure homogeneity. The prepared solutions were then protected from light by wrapping them in aluminum foil and stored at a controlled room temperature of 23 °C in a laboratory cabinet. To stabilize the solutions, they were left to stand for 24 h before testing in laboratory experiments.

### 2.2. PINQ System Setup and Configuration

**Figure 1:**
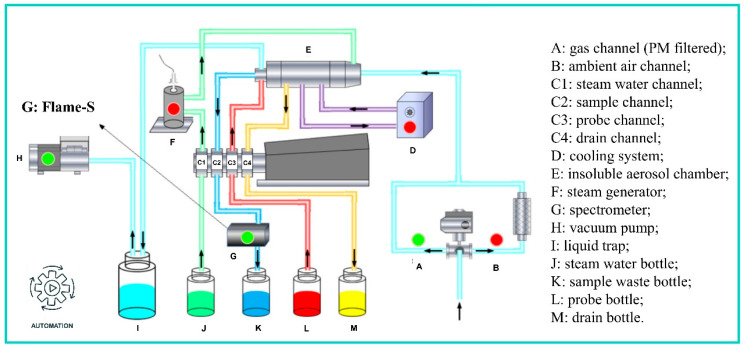
Diagram of the new PINQ system.

The new PINQ instrument was redesigned at Jinan University (model JCI ROS-1000, shown in Figure 1) based on a prototype developed by the Queensland University of Technology [25]. The original sampling system employed a combination of two sets of solenoid valves and pneumatic ball valves powered by an external gas cylinder. In the updated design, stainless steel three-way electric ball valves replace the original configuration, significantly reducing pipeline length and complexity. Furthermore, PLC-based unified control has been integrated, enabling full-component automation and preheating functionality. The spectrometer was upgraded from the USB2000+ (Ocean Optics Inc., Dunedin, FL, USA) to the FLAME-S (Ocean Optics Inc.), configured with a 100 μm slit, a 200 ms integration time, an averaging count of 5, and a logging time resolution of 1 s. Building on the USB series, the FLAME-S offers enhanced thermal stability and inter-instrument consistency. Additionally, the system allows users to replace the slit according to specific requirements, increasing application flexibility. The digital/manual integrated control module consists of a touch-screen display and a control box, overseeing the operation of all the subsystems, and providing precise control over the sampling, collection, and detection processes to ensure reliable ROS measurement.

### 2.3. Experimental Procedures

The new PINQ instrument was installed in the JNU-QUT Joint Laboratory for Air Quality Science and Management. The laboratory environment was carefully controlled, with the room temperature maintained at 23 °C, and the relative humidity kept at 78%. Fluorescent lamps served as the primary source of illumination in the lab throughout the experimental setup to ensure consistent lighting conditions.

During the sampling process, the PM_2.5_ cyclone first filters out particles larger than 2.5 µm, allowing PM_2.5_ and gaseous components to enter the system. The electric ball valve controls two separate flow paths. One of these paths is equipped with a particulate filter, permitting only gaseous components to proceed to the subsequent instruments. These two pathways alternate at a predetermined frequency to measure the total ROS and gas-phase ROS. The combination of the steam generator and insoluble aerosol chamber facilitates the dilution and growth of particles as they pass through, allowing them to flash mix and react with the probe vortex via the inner cyclone component. This process effectively captures ROS, which then enters the spectrometer for real-time fluorescence detection.

#### 2.3.1. Standard Curve

The standard curve represents the relationship between the measured response of a physical or chemical property and the concentration of a series of reference materials with known components. In this study, standard solutions were prepared using BPEAnit-Me at concentrations of 5, 10, 50, 75, 100, 150, and 200 nmol, with AR-grade pure DMSO serving as the blank control. The standard curve was generated using the response of the standard solutions, delivered via a peristaltic pump directly to the spectrometer with a flow rate of 1 mL/min, from low to high concentration. This setup ensured the accurate calibration of the system and provided a reliable basis for quantitative measurements.

#### 2.3.2. Baseline Noise and LOD

The baseline noise reflects the random fluctuations on the baseline during the instrument measurements. The method used to determine baseline noise in this test is as follows: When the instrument is in optimal operating condition, pure DMSO is introduced instead of the probe, and 60 consecutive measurements are conducted. The standard deviation is used to quantify the extent of the baseline signal fluctuations, as shown in Equation (1).

(1) $$\sigma =\sqrt{\frac{\sum_{i=1}^{N}\;(x_{i}-\bar{x}{)}^{2}}{N}}$$

where σ is the standard deviation in the spectrometer counts and *N* is the total number of data points in the sample. $x_{i}$ is the *i*-th data point in the database in the spectrometer counts and $\bar{x}$ is the average value of the data points in the spectrometer counts.

The limit of detection (LOD) refers to the lowest concentration of an analyte that can be reliably distinguished from baseline noise and is conventionally defined as three times the noise level, as recommended by the International Union of Pure and Applied Chemistry [34]. In this study, the LOD for the system was determined using three times the standard deviation of the signal from pure DMSO, ensuring consistency with prior methodologies [25].

#### 2.3.3. Accuracy and Precision

Accuracy refers to the degree to which a measurement aligns with the true value, reflecting systematic error. Precision, on the other hand, indicates the consistency of repeated measurements under identical conditions, highlighting the extent of random error. In this experiment, the accuracy and precision of the instrument were evaluated by using a series of BPEAnit-Me standard solutions from 5 to 200 nmol, low to high. Each concentration was measured at least 180 s to ensure reliability. Accuracy and precision were calculated based on the last 60 s of measurements across each tested concentration, as shown in Equations (2) and (3).

(2) $$\delta =\frac{\left|\bar{Y}-Y_{s}\right|}{Y_{s}}\times 100\%$$

where *δ* is the accuracy of the instrument under test, %; $\bar{Y}$ is the mean value of the measured concentration from multiple measurements, nmol; and *Y_S_* is the concentration of the standard BPEAnit-Me, nmol.

(3) $$RSD=\frac{\sqrt{\sum_{i=1}^{n}{\left(Y_{i}-\bar{Y}\right)}^{2}}}{\bar{Y}}\times 100\%$$

where *RSD* is the precision of the instrument under test, %; *Y_i_* is the measured concentration value from the *i*-th measurement, nmol; $\bar{Y}$ is the mean value of the measured concentrations from multiple measurements, nmol; and *n* is the total number of recorded data points.

#### 2.3.4. Startup Stability Time

A warm-up operation is crucial to ensure spectrometer stability and measurement accuracy prior to data collection. The instrument’s operating conditions can be categorized into two scenarios: cold start and warm start. A cold start refers to the time required for the instrument to achieve operational stability following a complete power shutdown, typically after prolonged inactivity, such as during field sampling or maintenance. In contrast, a warm start involves temporarily suspending instrument operation while components like the spectrometer and peristaltic pumps remain in standby mode, with the spectrometer retaining power and communication with the computer. Warm starts are commonly encountered during manual sampling or experimental condition adjustments.

In this study, both cold and warm starts were evaluated for their effects on stability and reproducibility. The cold start procedure involved shutting down the instrument entirely for 24 h before initiating measurements with the probe flowing. The warm start involved turning off the spectrometer and peristaltic pump for 24 h while keeping other components like the laser in standby mode, followed by signal measurements upon restart. Continuous measurements were recorded for 3 h under both scenarios to assess the instrument’s performance.

#### 2.3.5. 24-H Concentration Drift

The 24 h concentration drift refers to the deviation or fluctuation in the measurement results recorded by a detector operating under stable conditions without external interference over a 24 h period. Baseline drift is determined by running the detector with probe solution and recording changes in the baseline signal over 24 h to assess the instrument’s stability. In this experiment, a prepared BPEAnit probe was used following a cold start test, and the signal was continuously measured for 24 h under a controlled environment and without man-made operation to evaluate the stability of the probe-instrument system under standard conditions.

#### 2.3.6. System Residue

System residue refers to the phenomenon where a high analyte concentration from a previous sample results in residual analyte remaining in the analytical system. This can cause the analyte to appear in subsequent measurements, even when the subsequent sample does not contain the target analyte. In this experiment, after system stabilization, the fluorescence cell was rinsed with DMSO for 20 min. Following this, standard samples with a maximum concentration of 200 nmol were injected continuously for another 20 min. After completing the injections, the cell was rinsed again with DMSO for an additional 20 min. Signal changes throughout the process were recorded to evaluate the system’s response time and residual values.

### 2.4. PINQ Data Analysis

The BPEAnit probe’s fluorescent response is expressed as an increase in the equivalent nanomolar concentration of its methyl adduct (BPEAnit-Me). This conversion factor is obtained by calculating the slope of a calibration curve, which is derived from the fluorescence readings of BPEAnit-Me standards across a range of concentrations. For measurements using the PINQ system, the equivalent response is adjusted based on the ratio of the liquid supply flow rate to the aerosol sampling flow rate. This normalization yields a measurement of PM-bound ROS, expressed as the equivalent concentration of BPEAnit-Me per cubic meter of air, in units of nmol m^−3^, as shown in Equation (4).

(4) $$C_{ROS}=FR\cdot CF\cdot \frac{q_{ls}}{q_{A}}$$

In this context, $C_{ROS}$ represents the ROS concentration in nmol m^−3^, *FR* is the spectrometer’s fluorescence response measured in unitless counts, *CF* is the calibration factor obtained from the calibration curve and given in units of nmol L^−1^ counts^−1^, *q_ls_* denotes the liquid sample flow rate (in L min^−1^), and *q_A_* represents the aerosol flow rate (in m^3^ min^−1^) [25].

## 3. Results and Discussion

### 3.1. Standard Curve

Figure S1 presents the calibration curve obtained by plotting the ROS concentration (nmol) against the corresponding detector response in counts using the BPEAnit-Me probe in the PINQ system. The calibration curve demonstrates a highly linear relationship between the concentration and signal response, represented by the equation y = 59.70x + 982.91 with an R^2^ value of 0.9999. This strong linearity indicates a robust response of the PINQ system across the tested concentration range, providing a reliable quantitative measure for ROS concentrations in the atmosphere. This linearity is essential for quantitative applications, as it allows for the accurate interpolation of ROS concentrations based on detector readings. The slope with 59.70 counts/nmol of the line represents the sensitivity of the system to ROS concentration changes. A higher slope value indicates increased sensitivity, meaning that small changes in the ROS concentration result in significant changes in the detector response, enhancing the accuracy of low-concentration measurements. The intercept of 982.91 counts reflects the baseline response, indicating the effective lower limit of the signal, which corresponds to the initial state when the probe has not reacted at all and should be consistent with the baseline value of DMSO. The R^2^ value is 0.9999, which is very close to 1, and has been repeatedly validated in the subsequent tests, demonstrating the reliability and stability of the method. The high sensitivity and stable baseline response are crucial for understanding the oxidative potential in ambient air, making the system suitable for detecting trace levels of ROS in ambient air, which are often in the low nanomolar range.

### 3.2. Baseline Noise and LOD

Table S1 presents the baseline noise, 3σ ROS concentration, and LOD for the PINQ system using DMSO as the sample. These results provide a critical evaluation of the system’s sensitivity and baseline stability under theoretical conditions. In this study, the baseline noise was measured to be 9.63 counts, reflecting the high stability of the background signal in the PINQ system when no detectable ROS is present. This result lays the foundation for the reliable detection of low-concentration ROS. 3σ ROS concentration is calculated by applying three times the background noise into the calibration curve, the minimum detectable concentration was determined to be 0.48 nmol. This value highlights the high sensitivity of the PINQ system in detecting ROS, even at sub-nanomolar levels, making it suitable for atmospheric environments with low ROS concentrations. The LOD value of the PINQ system was determined to be 0.03 nmol by Equation (2), which is significantly improved compared to the previously reported value of 0.08 nmol for PINQ [25]. This improvement may be attributed to differences in the spectrometer model and parameter configuration. This highlights the substantial enhancement achieved with our newly developed PINQ system, which enhanced the PINQ system’s utility for the real-time, precise monitoring of oxidative species in atmospheric conditions.

### 3.3. Accuracy and Precision

**Figure 2:**
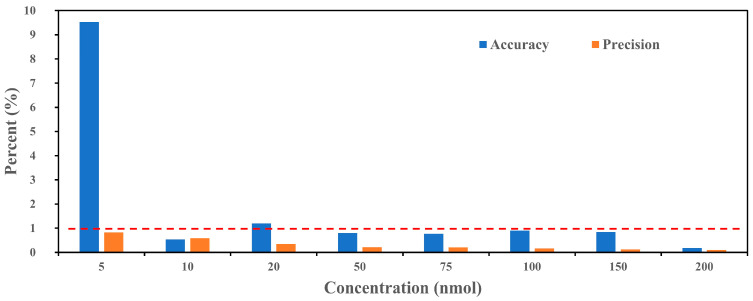
Accuracy and precision of the new PINQ system with various BPEAnit-Me concentrations (The blue bars represent accuracy, while the orange bars represent precision. The red dashed line marks the 1% threshold for both accuracy and precision).

Figure 2 illustrates the accuracy and precision of the PINQ system with BPEAnit across various ROS concentrations, ranging from 5 to 200 nmol. Accuracy (blue bars) and precision (orange bars) are presented as percentages, with a red dashed line indicating a 1% threshold. At low concentrations, particularly 5 nmol, the accuracy percentage is notably higher (~9%) compared to the other concentrations. This increased deviation is likely due to a combination of baseline noise and reduced signal–noise ratio at trace concentrations. As the ROS concentration increases, accuracy improves significantly, falling below the 1% threshold for concentrations of 50 nmol and above. This trend indicates that the PINQ system performs more reliably at mid-to-high concentrations, where the influence of baseline noise is minimal. For practical applications, this suggests that while the system is capable of detecting low ROS levels, the results at concentrations near the detection limit should be interpreted with caution due to higher variability. Precision, as indicated by the orange bars, remains consistently low (below 1%) across all the concentrations, including the lowest concentration of 5 nmol. This demonstrates the system’s strong reproducibility in repeated measurements, even under conditions where accuracy is slightly affected by baseline noise. The low precision values indicate minimal fluctuation in signal readings for the same concentration, highlighting the system’s reliability for continuous or long-term monitoring.

### 3.4. Startup Stability Time

**Figure 3:**
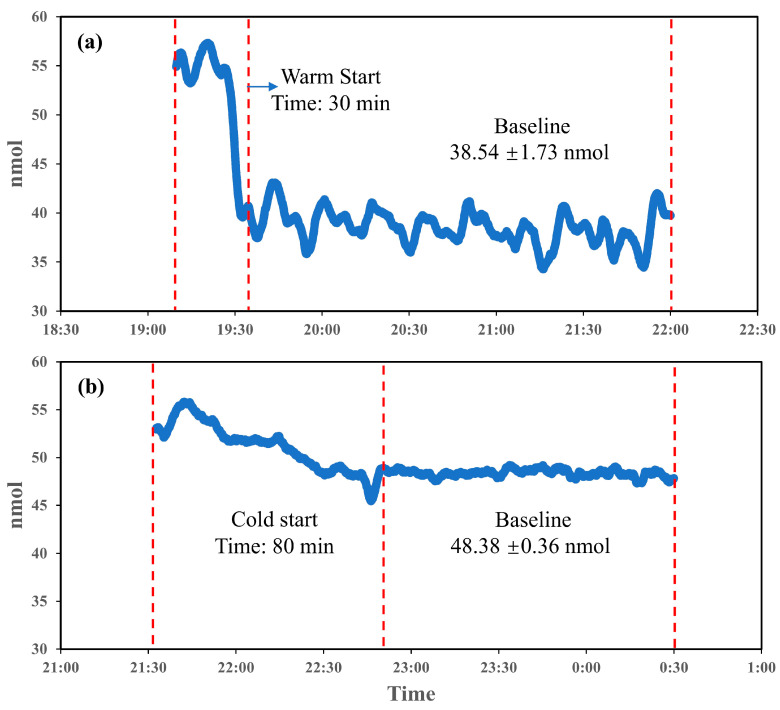
Startup stability time for PINQ ((**a**). warm start; (**b**). cold start).

Startup stability time is a critical prerequisite for all experiments involving the PINQ system. To evaluate the system’s startup stability, two conditions were examined: warm start and cold start, as illustrated in Figure 3a,b. During a warm start, the system reached stability within approximately 30 min, with an initial phase of minor fluctuations in the measurement values. Once stabilized, the baseline readings averaged 38.54 nmol with fluctuations of 1.73 nmol, indicating the effective preservation of baseline conditions. This rapid stabilization demonstrates the suitability of warm starts for scenarios requiring the frequent or rapid initiation of measurements, minimizing delays and enabling consistent data collection shortly after activation. However, an inherent issue was observed: the baseline value of the probe in the absence of sampling was greater than zero due to the weak fluorescence and self-oxidation of the probe.

In contrast, the cold start scenario in Figure 3b required a significantly longer stabilization period of approximately 80 min to achieve a stable baseline. The extended instability period reflects the system’s adjustment process from a completely inactive state, likely influenced by internal factors such as temperature variation among components. Once stabilized, the baseline demonstrated superior consistency, with fluctuations of only 0.36 nmol, markedly lower than the 1.73 nmol observed in the warm start. This improved stability is beneficial for detecting low-concentration and sensitive samples. However, the baseline value after a cold start was approximately 10 nmol higher than that observed in the warm start, further indicating the impact of probe self-oxidation on baseline elevation.

The comparison between the warm and cold starts underscores the operational trade-offs of each approach. Warm starts provide rapid stabilization, making them suitable for real-time and on-demand ROS monitoring in dynamic environments requiring frequent start-stop cycles. Cold starts, while requiring nearly three times the stabilization time, offer a more stable and low-variability baseline, enhancing reliability for sensitive measurements. Consequently, all the subsequent experiments were conducted following the cold start procedure to ensure consistent and stable experimental data.

### 3.5. 24-H Concentration Drift

**Figure 4:**
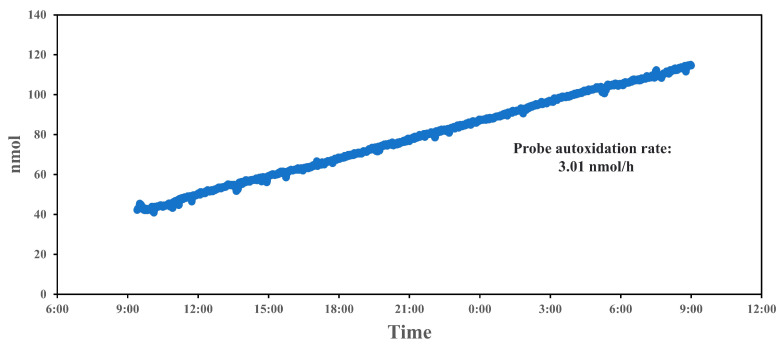
The 24 h concentration drift for BPEAnit in the PINQ system.

Over the 24 h period in Figure 4, a gradual increase in the fluorescence count readings is observed, indicating a positive drift in signal intensity. This upward trend suggests that the system experiences a baseline drift, possibly due to the cumulative auto-oxidation of the probe (BPEAnit) over time. The drift remains relatively steady, without abrupt changes, which implies that the system’s response to ROS does not fluctuate significantly but gradually increases due to the inherent chemical changes within the probe. The calculated auto-oxidation rate of 3.01 nmol/h reflects the rate at which the baseline signal increases independently of ROS concentration changes in the environment. Auto-oxidation is a common issue in ROS detection, as the probe itself can undergo oxidation reactions, leading to an elevated background signal. In this case, the linear increase in counts suggests that BPEAnit is susceptible to auto-oxidation, contributing to the observed signal drift, which explains the baseline change in the following tests. This steady rate provides a baseline correction factor that can be applied during data analysis to adjust for probe degradation over time, enhancing the accuracy of long-term ROS measurements. To eliminate and mitigate probe drift caused by auto-oxidation, we can optimize the system from both probe composition and experimental design perspectives. For the probe solution, incorporating appropriate stabilizers can help reduce auto-oxidation. Further investigations into the probe’s molecular structure, such as modifying functional groups, may enhance its intrinsic stability. For the experimental design, optimizing storage and handling conditions (e.g., controlling temperature and light exposure) can prevent unfavorable conditions that accelerate auto-oxidation. Additionally, implementing regular calibration and blank corrections, such as periodically subtracting background signals during experiments, enables effective drift detection and ensures the accuracy of the results under prevailing conditions. In addition, a stable auto-oxidation rate can be used to calculate the usable lifetime of the probe solution or to determine the appropriate initial concentration of the probe based on the required testing duration. Quantitative work on probe drift provides insight into the stability of the PINQ system over extended measurement periods, which is crucial for long-term atmospheric ROS monitoring.

### 3.6. System Residue

**Figure 5:**
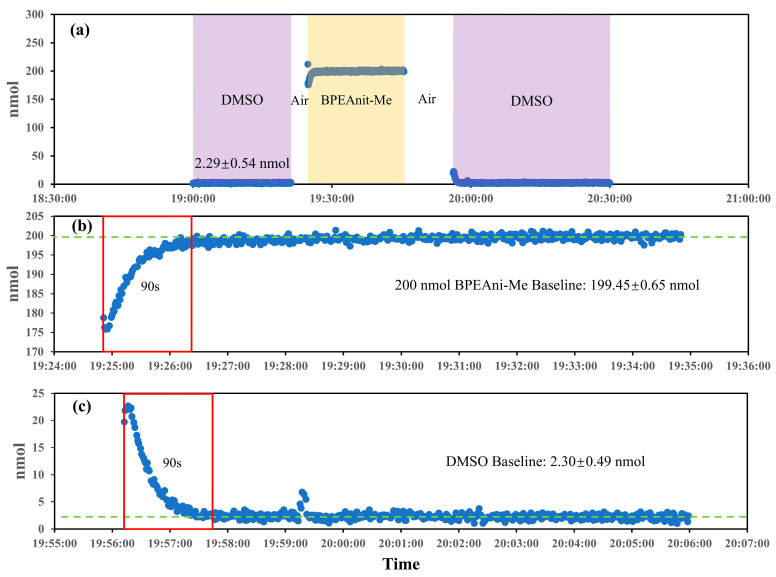
System residue evaluation in the new PINQ system using the DMSO and BPEAnit-Me probes. ((**a**). Baseline signal changes in sequential transitions between DMSO, air, and 200 nmol BPEAnit-Me; (**b**). response time and baseline for BPEAnit-Me after DMSO exposure; (**c**). response time and baseline for DMSO after high-concentration exposure).

The system residue was evaluated by monitoring the PINQ system’s response during transitions between DMSO, air, and 200 nmol BPEAnit-Me, as illustrated in Figure 5. The results provided insight into the system’s baseline behavior, residue clearance, and response stability. As shown in Figure 5a, the DMSO baseline stabilized at 2.29 ± 0.54 nmol, reflecting the system’s background signal in the absence of ROS; subsequently, 200 nmol BPEAnit-Me reached a steady state after a brief rise. The details in Figure 5b highlight the response stability for high concentrations of BPEAnit-Me. The system reached a stable signal within 90 s and maintained consistency with a low standard deviation (±0.65 nmol), demonstrating the fast response time and excellent precision for ROS detection at higher concentrations. Similarly, when switched to DMSO, the baseline returned to 2.30 ± 0.49 nmol after the flushing in Figure 5c, with stabilization also occurring within 90 s, which is almost the same as the values obtained before the high-concentration test, representing the consistency of mixing time and residue clearance time and proving that the effect of short-term high-concentration sample on the system can be ignored after cleaning. This test reflects the system’s capability to rapidly clear residual probes and maintain a low, stable baseline, ensure accurate and repeatable ROS measurements, and make the system suitable for real-time and long-term monitoring applications in diverse environmental conditions.

### 3.7. Comparison with Other Methods

To evaluate the performance of the PINQ system with the BPEAnit probe, it is essential to compare its sensitivity, accuracy, and operational convenience with commonly used ROS detection methods, such as the DTT assay and DCFH-DA method. Each method has distinct characteristics in terms of sensitivity, ease of operation, response time, and adaptability to environmental measurements, which are crucial for atmospheric ROS monitoring.

It is noteworthy that startup stability time has not been reported in the existing literature on ROS detection instruments. According to the findings from this study, startup stability is a crucial aspect of quality control, and there are significant differences in the application scenarios of warm and cold starts. The application scenarios for startup procedures are recommended to measure and describe across different methods and environments in future studies.

Compared with the latest studies, it can be observed that the R^2^ values of the standard curves for most online ROS measurement devices are above 0.99 [3,35,36,37,38,39,40,41]. Among them, the R^2^ value of OPROSI reaches as high as 0.9999 [36], which is consistent with the findings of this study, making it one of the most responsive methodologies currently available. Since DTT employs an absorbance-based method, its response slope cannot be directly compared with that of BPEAnit. DCFH, which uses a fluorescence spectroscopy method similar to the one in this study, allows for direct comparison after converting the signal to nmol-based slopes. It was found that the slope for ROS sampling analysis is 0.5 counts/nmol [35], OPROSI is 0.5 counts/nmol [36], PINQ-QUT is 4.69 counts/nmol [3], and online semi-continuous ROS instrument is 34.4 counts/nmol [42], all of which are lower than the slope of 59.70 counts/nmol obtained in this study, demonstrating the superior sensitivity of our system for ROS detection.

Regarding the comparison of LOD, Reece et al. conducted a detailed comparison previously [25]. This system is based on this prototype, incorporating a new spectrometer and an optimized structure, which further reduced the LOD from 0.08 to 0.03 nM/m^3^. However, the experimental results revealed that BPEAnit exhibits a similar self-oxidation behavior as DCFH, contrary to the previous assumption that BPEAnit would not undergo self-oxidation [25]. As observed, the system exhibits a steady auto-oxidation rate of 3.01 nmol/h, allowing predictable corrections over time. In contrast, the DTT assay often suffers from instability over prolonged measurements, particularly due to its interaction with transition metals, which can cause unaccounted drift [43,44]. The DCFH-DA assay is also prone to baseline instability, especially under variable temperature and humidity conditions, leading to inconsistent results if not frequently recalibrated. It has been shown to exhibit nonlinear drift both at room temperature and under refrigerated conditions [45]. The PINQ system’s predictable drift rate enhances its utility for long-term monitoring, as it allows for systematic baseline adjustments, reducing the risk of data inaccuracies in continuous applications.

System residue has been a concern in methods like DCFH and DTT, as probe residue can increase the baseline values [24]. Insufficient washing can lead to residual fluorescence, complicating data interpretation [46]. Self-cleaning procedures have already been implemented in some instrument testing [47,48]. However, the extent of system residue and the required cleaning time for the probe during ROS measurements have not been quantified in previous studies. In this study, we verified the inherent response time of the PINQ-BPEAnit system as well as the extremely low residual rate during short-term high-concentration sampling, which holds significant guidance for testing short-term low-concentration samples.

These advantages position the PINQ-BPEAnit system as a robust tool after standardized operations for atmospheric ROS monitoring, providing reliable and consistent data for both research and policy-driven applications. Its capability for continuous, real-time analysis with high sensitivity makes it valuable for studies focusing on the health impacts of ambient ROS levels.

## 4. Conclusions and Implication

To evaluate the reliability and limitations of the PINQ with BPEAnit as a ROS detection probe, we rebuilt the new PINQ system and standardized its operation process. The system demonstrates excellent sensitivity, with a limit of detection (LOD) of 0.03 nmol·m^−3^, a high response slope of 59.70 counts/nmol, and strong linearity (R^2^ = 0.9999). Moreover, all the tested concentration levels showed good accuracy and precision, confirming the systems robust performance for detecting trace levels of ROS in the atmosphere. It should be noted that the accuracy at the 5 nmol concentration reached 9.53%, which, while marginally lower than the general requirement of 10%, indicates that the baseline fluctuations may have a greater impact on low-concentration samples.

Building on traditional QA/QC practices, we tested more standardized procedures. Startup stability time is a critical experimental prerequisite that significantly affects the effectiveness and repeatability of short-term experiments. While a warm start requires only 30 min, it exhibits weaker stability, which may adversely impact tests involving low concentrations. In contrast, although a cold start takes a longer time (80 min), it provides superior baseline stability, with fluctuation reduced to one-fifth of those observed during a warm start.

During the experiments, we observed the auto-oxidation of the BPEAnit probe. Testing the 24 h drift under laboratory conditions revealed a linear growth in the probe signal, with an auto-oxidation rate of 3.01 nmol·m^−3^ per hour. This metric is crucial for long-term, real-time monitoring and for establishing standardized protocols.

Additionally, we investigated the potential effects of system residues on the baseline and found that short-term exposure to high-concentration samples within the standard curve range does not affect the baseline. The consistent stabilization time of 90 s further highlights an important parameter for future standardization efforts.

The PINQ system with the BPEAnit probe represents an improved method in ROS detection technology, offering reliable, real-time data with operational ease. Its application has the potential to improve atmospheric ROS monitoring practices, contributing to a better understanding of air pollution impacts on both the environment and public health. This study provides practical guidance, outlines the process of standardized use, and identifies the potential limitations.

Routine QA/QC procedures, such as daily testing and daily calibration, are essential and recommended. However, due to variations in the spectrometer and instrument, inconsistencies in probe types and batches, and changes in experimental conditions, significant differences in system testing parameters can occur, which highlights the necessity of additional standardization procedures like startup stability time, 24 h drift, and system residue. Further development could focus on enhancing the stability and sensitivity of the BPEAnit probe to minimize baseline drift, potentially through probe modification or improvements in system design. Expanding the calibration range to detect even lower ROS concentrations would increase its applicability, particularly in low-pollution environments or indoor air quality studies. Moreover, testing the system under varying environmental conditions, such as extreme humidity or temperature fluctuations, is also essential to demonstrate its robustness and ensure reliable performance across diverse scenarios.

## Data Availability

The raw data supporting the conclusions of this article will be made available by the authors on request.

## Supplementary Materials

The following supporting information can be downloaded at: https://www.mdpi.com/article/10.3390/s25041129/s1, Figure S1: Standard curve for PINQ (Error bars are annotated in the figure and range from 9 to 14 counts); Table S1: Baseline noise and LOD.
