# Supplementary material for: Development and Validation of the Particle into Nitroxide Quencher System with BPEAnit Probe for High-Sensitivity Reactive Oxygen Species Detection in Atmospheric Monitoring
Source: Sensors (Basel). 2025 Feb 13;25(4):1129. doi: 10.3390/s25041129 (PMC11859408; doi:10.3390/s25041129)
Supplement: Supplementary file 1 [file sensors-25-01129-s001.zip › sensors-3423199-supplementary.pdf]

Supporting Information for

# Development and Validation of the Particle into Nitroxide Quencher System with BPEAnit Probe for High-Sensitivity Reactive Oxygen Species Detection in Atmospheric Monitoring

Ruiwen Wang[1,2,3], Jiawen Li[1,3], Hao Wang[1,3,4,*], Shuo Deng[1,3], Congrong He[2,3,4], Branka Miljevic[2,3,4] Zoran Ristovski[2,3,4,*], Boguang Wang[1,3,4]

[1] *College of Environment and Climate, Jinan University, Guangzhou 511443, China*

[2] *School of Earth and Atmospheric Sciences, International Laboratory for Air Quality and Health, Queensland University of Technology, Brisbane, QLD 4000, Australia*

[3] *JNU-QUT Joint Laboratory for Air Quality Science and Management, Jinan University, Guangzhou 511443, China*

[4] *Innovation base for air quality science and management for Guangdong, Hong Kong and Macao Greater Bay Area, Guangzhou 511443, China*

**Corresponding author:*

*Hao Wang (wanghao@jnu.edu.cn); Zoran Ristovski (z.ristovski@qut.edu.au)*

**Contents of this file**

Figure S1 and Table S1

**Introduction**

This supporting information is shown to support the scientific conclusions in the manuscript.

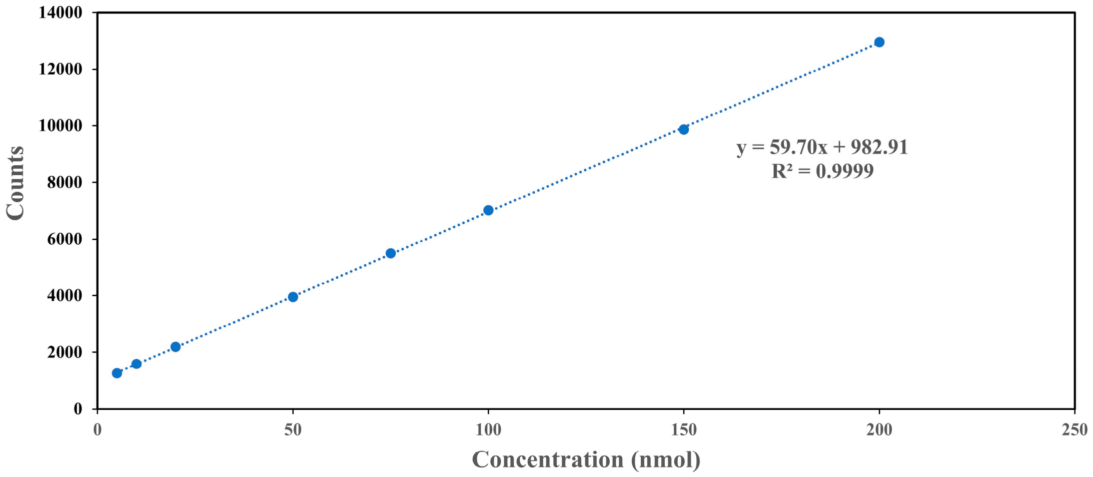


Figure S1 Standard curve for PINQ (Error bars are annotated in the figure and range from 9 to 14 counts)

Table S1 Baseline noise and LOD

| Samples (n=60) | Baseline noise (Counts) | 3σ ROS concentration (nmol) | PINQ LOD (nmol·m$^{-3}$) |
|---|---|---|---|
| DMSO | 9.63 | 0.48 | 0.03 |
